# Validation of a novel imaging approach using multi-slice CT and cone-beam CT to follow-up on condylar remodeling after bimaxillary surgery

**DOI:** 10.1038/ijos.2017.22

**Published:** 2017-07-14

**Authors:** Laura Ferreira Pinheiro Nicolielo, Jeroen Van Dessel, Eman Shaheen, Carolina Letelier, Marina Codari, Constantinus Politis, Ivo Lambrichts, Reinhilde Jacobs

**Affiliations:** 1 OMFS-IMPATH research group, Department of Imaging and Pathology, Faculty of Medicine,University of Leuven and Maxillofacial Surgery Department, University Hospitals Leuven, Leuven, Belgium; 2 Department of Oral and Maxillofacial Radiology, University of los Andes, Santiago, Chile; 3 Unit of Radiology, IRCCS Policlinico San Donato, Via Morandi 30, San Donato Milanese, Milan, Italy; 4 Department of Morphology, Faculty of Medicine, University of Hasselt, Campus, Diepenbeek, Belgium; 5 Department of Dental Medicine, Karolinska Institutet, Huddinge, Sweden

**Keywords:** condylar resorption, cone-beam computed tomography, mandibular condyle, multi-slice computed tomography, three-dimensional imaging

## Abstract

The main goal of this study was to introduce a novel three-dimensional procedure to objectively quantify both inner and outer condylar remodelling on preoperative multi-slice computed tomography (MSCT) and postoperative cone-beam computed tomography (CBCT) images. Second, the reliability and accuracy of this condylar volume quantification method was assessed. The mandibles of 20 patients (11 female and 9 male) who underwent bimaxillary surgery were semi-automatically extracted from MSCT/CBCT scans and rendered in 3D. The resulting condyles were spatially matched by using an anatomical landmark-based registration procedure. A standardized sphere was created around each condyle, and the condylar bone volume within this selected region of interest was automatically calculated. To investigate the reproducibility of the method, inter- and intra-observer reliability was calculated for assessments made by two experienced radiologists twice five months apart in a set of ten randomly selected patients. To test the accuracy of the bone segmentation, the inner and outer bone structures of one dry mandible, scanned according to the clinical set-up, were compared with the gold standard, micro-CT. Thirty-eight condyles showed a significant (*P*<0.05) mean bone volume decrease of 26.4%±11.4% (502.9 mm^3^±268.1 mm^3^). No significant effects of side, sex or age were found. Good to excellent (ICC>0.6) intra- and inter-observer reliability was observed for both MSCT and CBCT. Moreover, the bone segmentation accuracy was less than one voxel (0.4 mm) for MSCT (0.3 mm±0.2 mm) and CBCT (0.4 mm±0.3 mm), thus indicating the clinical potential of this method for objective follow-up in pathological condylar resorption.

## Introduction

Orthognathic surgery often creates changes in the location of the temporomandibular joint (TMJ). These positional alterations may induce functional stress on the mandibular head, thereby causing condylar remodelling, which is considered to be a possible aetiology of skeletal relapse after orthognathic surgery.^1^ When patients show clinical signs and symptoms of potential postsurgical condylar resorption, radiographic imaging is required to obtain additional diagnostic information to optimize patient treatment and to estimate the severity of the condition. High-resolution three-dimensional (3D) imaging is the standard radiographic evaluation tool. Magnetic resonance imaging is used to determine the actual position of the disc, while multi-slice computed tomography (MSCT) and Cone-Beam CT (CBCT) are used to evaluate osseous pathological changes at the condylar level.^2^ MSCT is often the preferred imaging modality to plan orthognathic surgery, owing to its high contrast-to-noise-ratio. However, if a follow-up of the surgical stability is indicated, the patient is exposed to a high dose of radiation. Therefore, an alternative low-dose CBCT approach can be recommended. Recent advancements in CBCT technology have allowed sufficient resolution to accurately depict bone structures and render 3D models.^3, 4^ Unfortunately, this potential is not fully exploited in the routine dental practice. Most of the time, methods to assess condylar resorption remain limited to two-dimensional (2D) measurements.^5, 6, 7, 8, 9^ Or, when in 3D, inner trabecular structure is neglected.^10, 11, 12, 13, 14, 15^ To properly follow-up condylar changes over time, a precise and reliable diagnostic tool is mandatory.

Therefore, the goal of the present study was to introduce a new 3D procedure to objectively quantify condylar remodelling in MSCT and CBCT images. As a second objective, the reliability and accuracy of this condylar volume quantification were evaluated.

## Materials and methods

### Clinical assessment of condylar remodelling

#### Data acquisition

Twenty patients (11 female and 9 male; mean age±standard deviations: 23±10) who underwent bimaxillary surgery at the department of Oral and Maxillofacial Surgery (University Hospital Leuven, Leuven, Belgium) and presented signs and symptoms of temporomandibular joint dysfunction (TMD) were retrospectively included in the study. All patients provided informed consent, and ethical approval was obtained from the medical ethics committee of University Hospitals KU Leuven, Leuven Belgium (S57587). All patients were orthodontically treated before and after surgery. No subjects had a previous history of maxillofacial trauma or any known autoimmune or metabolic bone disease.

Following the clinical protocol at our institution, preoperative images were acquired with Somatom Definition Flash MSCT (Siemens Healthcare, Erlangen, Germany) by using a high-resolution (400 μm) scanning protocol with the following exposure settings: 120 kVp, 250 mA, U75 kernel and a 500 × 500 mm field of view (FOV). The voxel size of the MSCT was not isotropic, with a slice thickness of 500 μm. Low-dose ProMax 3D Max CBCT (Planmeca Oy, Helsinki, Finland) was used for the postoperative follow-up. All of the MSCT and CBCT scans were taken with a wax bite to ensure that the mandible was in a centric position.^16^ A scout view was taken before the scan in order to include both condyles without truncation artefacts. Thereafter, a large 230 × 260 mm FOV scan was acquired at 96 kVp, 5 mA and 400 μm resolution.

#### Image analysis

**Figure 1:**
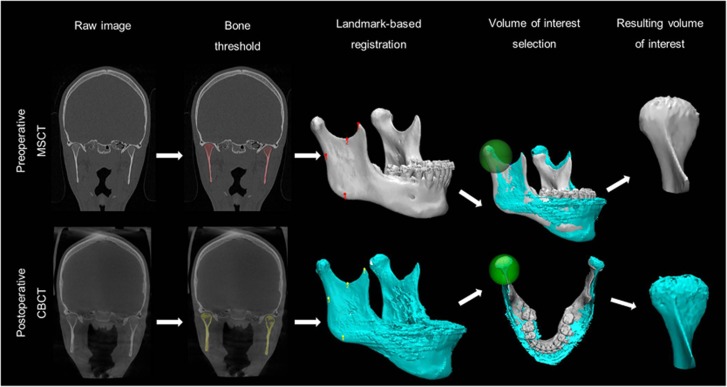
**Workflow of the patient data image analysis**. CBCT, cone-beam computed tomography; MSCT, multi-slice computed tomography.

Preoperative MSCT and postoperative CBCT images underwent the same image processing procedure shown in Figure 1. Each MSCT and CBCT scan in DICOM format were imported into Mimics medical image processing software (Version 18.0, Materialise, Leuven, Belgium), and were resliced to an isotropic voxel dimension of 400 μm^3^. The mandibular bone was semi-automatically delineated by using a global threshold algorithm. The computer suggested bone threshold values were visually confirmed in order to allow for the best segmentation overlap with the original image. The segmented mandibles were rendered in 3D and saved in.stl format for further image processing purposes. Subsequently, the pre- and postoperative 3D models were imported in 3-Matic software (Version 9.0, Materialise, Leuven, Belgium), and a minimum of three anatomical landmarks per mandible were chosen by an experienced radiologist (LFPN). The anatomical landmarks were selected on the coronoid process and the mandibular ramus and angle, owing to expected changes in the mandibular body and symphysis morphology after orthognathic surgery and the presence of metal braces from orthodontic treatment. On the basis of the best fit of these landmarks, the computer calculated the optimal translation and rotation between the pre- and postoperative 3D mandibles by minimizing the mean square distance between the coronoid and ramus surfaces. In this way, the left and right condyles were registered separately. To achieve uniform selection of each condyle from the mandible, a standardized sphere with its border passing through the lowest point of the mandibular notch was created around the condyle. After consistent extraction of the condyle, the condylar bone volume was automatically calculated in mm^3^.

### Validation of method reproducibility

**Figure 2:**
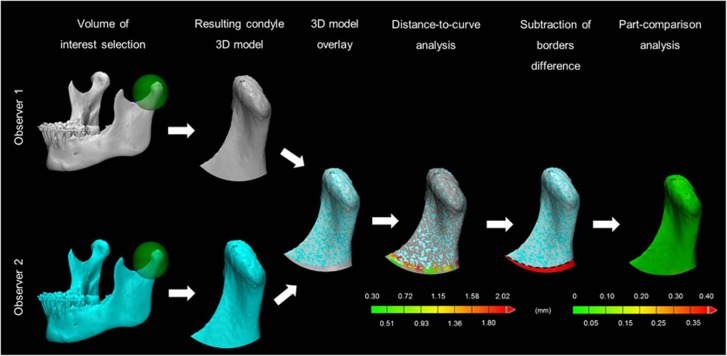
**Workflow of the method reproducibility with MSCT data.** 3D models generated by different observers were overlaid. A distance-to-curve analysis detected differences in the region of interest selection (mean: 1.25 mm, range: 0.3–2 mm). Before the part-comparison analysis, the error generated by the region of interest selection is subtracted (red colour). In this way, the calculation of the local differences between the 3D models is possible without counting the error of the region of interest selection. Part-comparison analysis from presented case shows a mean distance of 0.1 mm between the two condyles in the 3D models. 3D, three-dimensional; MSCT, multi-slice computed tomography.

The reproducibility of the condylar volume determination may be affected by subjective VOI and bone threshold selections. Therefore, inter- and intra-observer reliability were calculated between assessments made by two experienced radiologists (LFPN and CL) at two time points with a 5-month interval (T1 and T2) in a set of 10 randomly selected patients. The MSCT and CBCT data were thresholded by each observer, and 3D models were generated on the basis of the individual segmented images. A VOI selection procedure was performed according to the clinical assessment described above. The resulting 3D models of the corresponding condyles selected by each observer were overlaid to calculate the discrepancies between both models using distance-to-curve and part-comparison analyses (Figure 2). First, the mean distance (in mm) between the lower borders was automatically quantified and used as a measurement of discrepancy in VOI selection. Second, the shortest distance (in mm) between each internal and surface part of the two models was automatically calculated after the removal of dissimilarities in VOI selection through model subtraction and used as a measurement of threshold selection discrepancy.

### Validation of condylar mineralized bone assessment

To examine the accuracy of the analytic approach, one dry human mandible was obtained from the Institute for Biomedical Research, Hasselt University and was approved for research by the ethical committee of the University Hospitals KU Leuven (S55619). The same scan settings were used according to the clinical scanning protocol for TMJ visualization with Somatom Definition Flash MSCT and ProMax 3D Max CBCT.

**Figure 3:**
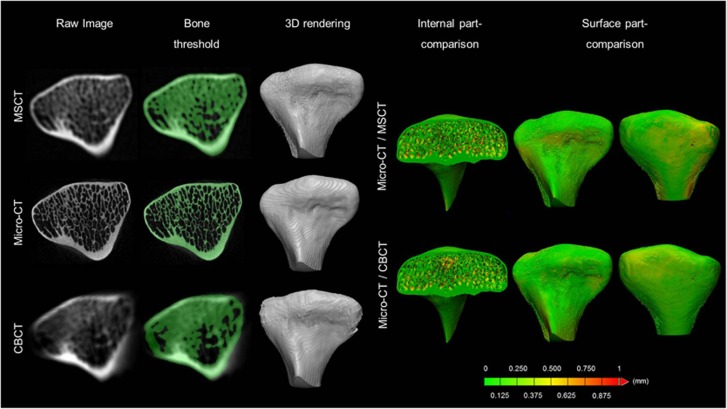
**Accuracy of the condyle mineralized bone assessment.** First column: The results of image registration between the different modalities. Second column: Image segmentation based on grey values. Third column: rendered 3D condylar models for all imaging modalities. The part-comparison analysis colour-codes the amount of overestimation in the inner and outer (surface) bone quantity between MSCT/CBCT and micro-CT. The green colour indicates an overestimation of less than one voxel between both 3D models and yellow and red indicate an overestimation of more than 0.375 mm. 3D, three-dimensional; CBCT, cone-beam computed tomography; MSCT, multi-slice computed tomography.

The left condyle was sectioned 1 cm below the lowest point of the sigmoid notch to allow micro-CT (SkyScan 1172, SkyScan, Kontich, Belgium) scanning, and further served as the gold standard for accuracy measurements. A high-resolution (35 μm) scan protocol was used at 100 kVp, 100 μmA, 1 mm aluminium, 180° rotation with an angular step of 0.7° and a frame averaging of 6, thus resulting in a total scan time of 9 min. The image stacks were reconstructed with an isotropic voxel size of 35 μm^3^ in NRecon software (version 1.6.5, Bruker micro-CT), which were used for further image analysis. An overview of the image processing steps is shown in Figure 3. The acquired CBCT and MSCT images were spatially aligned with the corresponding micro-CT images by using a mutual information algorithm.^17^

After precise registration, each image was semi-automatically segmented and 3D rendered by using the same processing protocol as in the clinical evaluation. The mineralized condylar bone volume was automatically calculated and compared among the different imaging modalities. A more detailed part-comparison analysis was conducted to evaluate the structural dissimilarity of the inner and outer mineralized condylar bone between MSCT/CBCT and micro-CT images.

### Statistical analysis

The sample size was calculated by using a postoperative reduction in condylar volume of 105 mm^3^±90 mm^3^ after bilateral sagittal split advancement osteotomy, which was obtained from a previous study.^14^ A power analysis in G*Power 3.1 suggested a sample size of 11 patients assuming 95% power with an α of 0.05.^18^ A repeated measures ANCOVA was used to examine the effects of time (preoperative MSCT/postoperative CBCT) and side (left/right) as within-subject factors on condylar volume (in mm^3^). Sex and age were included as covariates. The intra-class correlation coefficient (ICC) was calculated between condylar volume, as a measure to evaluate the agreement within and between the observers. The two-way mixed single measures for consistency were reported. The reproducibility of the procedure was influenced by the differences in VOI selection and bone threshold selection. Measurement discrepancies and standard deviations were reported for condylar volume (in mm^3^), VOI selection (in mm) and bone threshold selection (in mm). A one-way ANOVA was used to examine condylar volume differences between MSCT, CBCT and the gold standard, micro-CT.

The statistical analysis was conducted in IBM SPSS statistical software (Version 22.0, IBM, New York, USA). The significance level α was set for all statistical tests at 0.05.

## Results

### Clinical assessment of condylar remodelling

The 40 condyles of 20 patients were analysed. All condyles, except for two from the same patient, exhibited remodelling of the mineralized volume of the condyle ranging up to a maximum of 46.5% (1 088.7 mm^3^) with a mean of 26.4%±11.4% (502.9 mm^3^±268.1 mm^3^). Both the left and right condyles exhibited significant (*P*<0.001) postoperative condylar remodelling volumes compared with their preoperative volumes. In individual subjects, differences in volumetric remodelling between the left and right condyles varied from 0.05% (4.8 mm^3^) to 14.9% (267.3 mm^3^) with a mean±standard deviations of 6.9%±4.8% (114.4 mm^3^±94.9 mm^3^), with no statistically significant difference (*P*=0.55). No significant sex effect was observed in the present sample. In females, the condylar remodelling volume decreased on average by 27.0%±13.4% (470.4 mm^3^±301.1 mm^3^), and in males, it decreased by 25.7%±8.8% (542.6 mm^3^±223.3 mm^3^). Two condyles in one patient showed an increased volume of 7% (73.4 mm^3^) and 11% (105.0 mm^3^) in the left and right sides, respectively.

### Validation of method reproducibility

Excellent evaluation reliability was obtained between observers at T2 (ICC=0.93 for MSCT; ICC=0.91 for CBCT) and within observer 1 (ICC=0.96 for MSCT; ICC=0.89 for CBCT). ICC values were excellent and good within observer 2 (ICC=0.96 for MSCT; ICC=0.73 for CBCT) and between observers at T1 (ICC=0.89 for MSCT; ICC=0.6 for CBCT). Volume, VOI and segmentation discrepancies are shown in Table 1.

### Validation of condylar mineralized bone assessment

The micro-CT showed a lower condylar volume (1 167 mm^3^) compared with MSCT (1 834 mm^3^) and CBCT (1 732 mm^3^). The part-comparison analysis indicated an overestimation of the bone segmentation of 0.3 mm±0.2 mm for MSCT and 0.4 mm±0.3 mm for CBCT.

## Discussion

In the present study, preoperative MSCT data and low-dose CBCT data at the 6-month postoperative follow-up from patients who underwent bimaxillary surgery were used to objectively assess condylar volume changes and to validate a new quantification method for condylar volume. The present method showed bone remodelling in 95% (38/40) of the condyles with an average of 26.4% mineralized bone loss. The reproducibility between the two observers was good to excellent and the accuracy with micro-CT indicated a bone segmentation overestimation of <0.4 mm on average, corresponding to less than one voxel.

The reported incidence of condylar resorption after orthognathic surgery ranges from 1% to 31% depending on the defined criteria and various surgical and non-surgical risk factors.^19, 20, 21^ Young age and female sex are two of the most common patient-related risk factors for postoperative condylar resorption, mainly occurring in young female individuals in the second and third decades of life.^14, 19, 21, 22^ In this study, no correlations between age, sex and condylar alterations were found, although more females in that age range were included. Unfortunately, the assessment of the age predilection is complicated, because most orthognathic surgery is performed in young patients. The female predisposition to condylar resorption, which occurs at a frequency of 9:1,^19, 22^ has been suggested to be related to a regulatory effect of oestrogen on bone metabolism in the TMJ. Moreover, as an important consideration, women seek medical help for dentofacial abnormalities more often than men.

Because of the study design and the implementation of only clinical and two-dimensional radiological data for the diagnosis of condylar alterations, most published studies have intrinsic limitations, although the diagnosis of condylar resorption in longitudinal studies is often based on a qualitative assessment of the mandibular condyles on OPG.^23^ The major advantage of CT modalities compared with conventional radiographs is the possibility to render 3D models, thus allowing for linear, angular and volumetric measurements of the facial skeleton.^24, 25^ The 3D rendering of the condyles has already been described in previous studies to follow-up on the condylar volume after orthognathic surgery.^10, 11, 14^ However, currently, no quantitative criteria for condylar bone loss has been accepted by the scientific community. In contrast with previous studies conducting follow-ups on surface and morphological condylar changes,^12, 24, 26^ this novel imaging procedure attempted to depict the overall mineralized bone content. The purpose was to assess resorption by quantifying the volume of mineralized bone, both in the cortical surface and in the trabecular bone. In this way, the present study found a greater condylar volume decrease compared with a previous study^14^ that has reported no more than a 6.1% decrease in the original condylar volume in 55% of the condyles one year after surgery. This contradiction may also be explained by the differences in the follow-up period. Six months is too short to determine the long-term effects of orthognathic surgery on condylar status, because the effects can develop for more than 1 year. The results of the physiological remodeling process, which initially starts with cortical demineralization, generate an early radiologic depiction that is unclear, even when a bony matrix with a low degree of mineralization would be present. Therefore, a subsequent remineralization process that enhances cortical visibility and the radiological measurable condylar volume over time is plausible. The present study focused on the assessment of condylar remodelling, which we hypothesized to be the first manifestation of a possible resorptive process.

Owing to differences in image acquisition and scan parameters, MSCT and CBCT generate images with different qualities, thereby influencing bone segmentation.^27^ In the current study, the bone structure was visually more accurate in MSCT, a result that may be explained by this modality’s higher contrast-to-noise ratio that favours bone segmentation. This segmentation was based on image thresholding and only voxels with bone intensities were selected on the basis of the image histogram. However, these intensities, expressed in grey values, may vary with artefacts generated from metal, movement of the patient, partial volume averaging and the selection of the tube voltage and current. These artefacts may lead to a greater identification error of the condylar contours and, consequently, to measurement errors.^28^ To follow-up on condylar bone changes over time and to produce a visually acceptable 3D rendering, these inaccuracies must be taken into account.

Validation was performed to quantify these inaccuracies through the verification of both the reproducibility and the accuracy of the current method. The first measure verified segmentation and VOI selection repeatability, and the second measure verified segmentation accuracy. The segmentation procedure was based on the global thresholding of the mineralized bone, which was already proven to be more accurate than manual delineation of the condyle.^29^ The accuracy of the 3D volume rendering was based on this step. Using a part-comparison analysis to detect regional differences, the segmentation error was 0.2 mm on average for the observer reproducibility, in agreement with previous studies,^10, 15^ and less than one voxel for the accuracy measurements in both MSCT and CBCT.^28^ This overestimation may influence the quantification results, because the literature has reported condylar resorptive changes of 0.4 mm^(ref. 30)^ on average and up to at least 1.5 mm^(ref. 12)^ after 1 year of orthognathic surgery. Although the present MSCT and CBCT protocols showed comparable overestimations, the standardization of bone alteration measurements across time requires the use of the same scanning modality and protocol. Therefore, counterbalanced image quality and radiation dose are important. CBCT is considered to deliver a lower radiation dose to the patient, but the full head protocol may deliver a radiation dose comparable to that of MSCT with some machines.^31, 32^ The total condylar volume calculation is a sum of the segmentation and VOI selection. In this study, the size of the sphere was determined in each case according to the condyle size (which varied from 14 to 19 mm in radius). It was defined as the minimal size needed to involve the full condyle while the border of the sphere simultaneously passed through the lowest point of the sigmoid notch. This could occur only by manually centralizing the sphere over the condyle. If the centre of the sphere shifted, the mandible head would be outside the sphere or the border would not pass through the lowest point of the sigmoid notch. According to our results, a higher discrepancy relative to VOI selection was observed compared with segmentation selection, especially with MSCT. VOI selection, which represents the total condyle volume, is a result of the anatomical marker choice, which is considered a reliable and reproducible anatomical marker that is not affected by natural growth or surgical interventions,^10^ with a 0.2-mm identification error.^15^ Although the identification of this anatomical point has a lower error, this error is distributed over the full condylar volume when placing the sphere around the condyle, thus potentially explaining the high variability in the distances between the limiting lower borders between the two models. In the clinical data, when comparing pre- and postoperative volumes, the VOI selection error can be overcome by the previous spatial alignment of the two condyles and the selection of the VOI simultaneously. In longitudinal studies, this spatial alignment can compensate for differences in the scanning head position and coordinate system, thus allowing for standardized measurements between images acquired at different time points. Currently, different methods are available to superimpose 3D images. Surface- and voxel-based registration have been reported to have similar accuracies in the assessment of surgical changes after orthognathic surgery.^33^ However, voxel-based registration relies on the grey-scale intensity of the DICOM image voxels, thus suggesting the need for more efficient computers and a longer processing time.^33^ This method may fail when superimposing two objects with significant morphological variability.^12, 13, 34^ After mandibular advancement, the ramus is the only anatomical part preserved from the osteotomy. Because the mandible width also changes after surgery, a simultaneous superimposition of both rami would not be possible without accounting for the mandible displacement. When landmark surface-based registration is applied, only one ramus can be superimposed at a time, and only differences in condylar volume can be assessed.

Our novel analysis method allows for the 3D quantification of the mineralized bone in the mandibular condyles, thus revealing differences between preoperative and postoperative situations. The proposed method may be of value during objective assessments and follow-ups of pathological condylar resorption after bimaxillary surgery.

## Figures and Tables

**Table 1:** The mean and standard deviations of the absolute discrepancy measurements of volume, VOI selection and segmentation between observers (inter) and within-observer (intra) in MSCT and in CBCT data

Scanner type	Observer relation	Volume/mm^3^	VOI/mm	Segmentation/mm
MSCT	Intra	120.0±92.6	0.9±0.8	0.1±0.2
	Inter	178.6±149.3	0.8±0.8	0.3±0.3
CBCT	Intra	136.1±143.5	0.5±0.5	0.2±0.2
	Inter	174.1±201.1	0.7±0.7	0.2±0.2

CBCT, cone-beam computed tomography; MSCT, multi-slice computed tomography; VOI, volume of interest.
